# Evidence that women meeting physical activity guidelines do not sit less: An observational inclinometry study

**DOI:** 10.1186/1479-5868-9-122

**Published:** 2012-10-04

**Authors:** Lynette L Craft, Theodore W Zderic, Susan M Gapstur, Erik H VanIterson, Danielle M Thomas, Juned Siddique, Marc T Hamilton

**Affiliations:** 1 Feinberg School of Medicine, Northwestern University, 680 N Lake Shore Drive, Suite 1400, Chicago, IL, 60611, USA; 2 Pennington Biomedical Research Center, 6400 Perkins Road, Baton Rouge, LA, 70808, USA; 3 American Cancer Society, 250 Williams Street, Atlanta, GA, 30303, USA; 4 Department of Preventive Medicine, Northwestern University, 680 N. Lake Shore Drive, Suite 1400, Chicago, IL, 60611, USA

**Keywords:** Inactivity physiology, Walking, Stepping, Standing, Non-exercise physical activity, Sedentary behavior

## Abstract

**Background:**

The inactivity physiology paradigm proposes that sedentary behaviors, including sitting too much, are independent of the type of physical activity delineated for health in the Physical Activity Guidelines for Americans. Thus, we hypothesized that, when accounting for behaviors across the entire day, variability in the amount of time spent sitting would be independent of the inter-and intra-individual time engaged in sustained moderate-to-vigorous physical activity (MVPA).

**Methods:**

Ninety-one healthy women, aged 40–75 years, completed a demographic questionnaire and assessment of height and weight. Participants wore the activPAL activity monitor for one week and time (minutes/day) spent sitting, standing, stepping, and in sustained bouts (bouts ≥10 minutes) of MVPA were quantified. The women were then stratified into groups based on weekly sustained MVPA. Additionally, each day of data collection for each participant was classified as either a “sufficient” MVPA day (≥ 30 min of MVPA) or an “insufficient” MVPA day for within-participant analyses.

**Results:**

Time spent sitting, standing, and in incidental non-exercise stepping averaged 64, 28, and 11 hrs/week, respectively, and did not differ between groups with individuals meeting/exceeding the current exercise recommendation of 150 min/week of sustained MVPA in ≥10 minutes bouts (M = 294 min/week, SD = 22) compared to those with none or minimal levels (M= 20min/week, SD = 4). Time spent sitting (M = 9.1 hr/day, SD = 0.19 vs. M = 8.8 hr/day, SD = 0.22), standing (M = 3.9 hr/day, SD = 0.16 vs. M = 3.9 hr/day, SD = 0.15), and in intermittent stepping (M = 1.6 hr/day, SD = 0.07 vs. M = 1.6 hr/day, SD = 0.06) did not differ between days with (~55 min/day) and without recommended MVPA.

**Conclusions:**

This study provides the first objective evidence that participation in sustained MVPA is unrelated to daily sitting duration in relatively healthy, middle and older-aged women. More research is needed to extend these findings to other populations and to inform distinct behavioral recommendations focused on sedentary time.

## 

Based upon decades of research, the public has come to understand the need for “aerobic exercise”. The current Physical Activity Guidelines for Americans (hereafter referred to as “guidelines”) call for the weekly accumulation of an equivalent of at least 150 min of moderate physical activity, sustained in bouts lasting 10 minutes or longer [1]. This type of sustained moderate-vigorous physical activity (MVPA) has thus been promoted as a healthy behavior positively affecting numerous health outcomes. Recently, the more ubiquitous types of sedentary behaviors (e.g., primarily sitting throughout the day) have been linked to deleterious health outcomes, independent of MVPA [2-5]. These recent findings necessitate the quantification of sedentary time and all non-exercise physical activity.

In spite of this independent relationship between sedentary behavior and health, few studies have examined the associations between objectively measured sedentary or sitting time and MVPA. The studies that have reported on this relationship have generally used self-report measures and have found equivocal associations [6-9]. One argument has been that exercise participation may inadvertently result in an *increase* in sedentary behavior, by reducing the drive to be active in non-exercise periods [10]. The opposite possibility is that adults who exercise regularly may generally have more energy, or have enhanced feelings of vigor, and *decrease* sedentary behavior [11,12]. Or, greater exercise time may simply displace sitting time [13]. Thus, one theoretical yet untested benefit of regular exercise is reduced sedentary time through more total upright activity. While such possibilities are plausible, objective evidence is necessary because there are likely many physiological and genetic determinants for subconscious spontaneous physical activity, and also because adults who make time to exercise may also make other healthy lifestyle choices favoring a less sedentary lifestyle [14]. Finally, it has been pointed out that it is hard to predict, without accurate measurements, how much exercise impacts all activity/inactivity over "the rest of the day" since so much time is spent in various sedentary behaviors and non-exercise physical activity. These behaviors include many hours of alternating between intermittent sitting and types of standing activity that do not involve much time walking, and thus are challenging to quantify without inclinometry and accelerometry.

Although not formally monitored in population health surveys, incidental low-intensity physical activity (LIPA) is of increasing interest in health research [15-18]; however, challenges in assessing changes in posture and low-intensity ambulatory movement have potentially contributed to the limited number of descriptive studies on this topic. Thus, the field presently lacks objective quantification of seated and upright postures throughout the day as assessed by wearable devices with inclinometry. Accordingly, the hypothesis raised in the second tenet of the inactivity physiology paradigm [3], that sitting and exercise-like behaviors are unrelated, independent, and not determined by the same factors, needs to be empirically tested with objective inclinometry [2-4,19-21]. Thus, there is a need to test this and the respected hypotheses described above [10,11,13,14] in order to know if meeting the guidelines for aerobic MVPA, with activities such as sustained brisk walking, either reduce, increase, or have no effect on the total daily sedentary time.

Hip-worn accelerometers [22-24] have been used to estimate the duration of sedentary time from total body movement. While accelerometers function well for many purposes, most models are not designed to accurately measure postures like sitting and standing. Although much research has gone into determining fixed acceleration cutpoints associated with various activity intensities, the fixed cutpoint method of estimating sedentary time (i.e., typically less than 100 counts/min from a commercially available monitor) was somewhat arbitrarily defined by researchers. Recent findings have raised concerns about how well this method actually measures sitting duration [25]. It is feasible to address these issues more definitively and overcome the limitations of traditional accelerometers with newer, validated wearable devices [25-27]. Consequently, in this study, we were able to objectively assess all sedentary and upright behaviors by using the activPAL monitor which utilizes both accelerometer and inclinometer functions. This device has been validated for the measurement of common behaviors such as sitting, standing, and walking [25-27].

The aim of the present study was to test the long-standing hypothesis [3,19] that the time spent performing exercise-like sustained MVPA vs. the time engaged in sedentary behavior (sitting) or total upright activity are distinct and unrelated. Put most simply, it is hypothesized that regular exercisers (people who meet or exceed guidelines for sustained MVPA) are not less sedentary. This could be the case if exercise does not reduce the total daily sitting time by a meaningful amount or influence the total daily physical activity (which is mostly LIPA). This proposition needs to be tested directly with quantitative methodologies. To the best of our knowledge, this hypothesis is yet untested using objective measures capable of quantifying all sitting, standing, sustained MVPA, and intermittent stepping over the entire waking day. To accomplish this, the present study has utilized accelerometer/inclinometer technology already validated for measuring postures [25] and stepping rate [26].

## Methods

### Participants

We recruited 100 women, aged 40–75 years, into the Food, Exercise, and Mammography study (FEMS). FEMS was a study examining relationships of lifestyle factors with mammographic breast density, as well the interrelationships among those factors. Participants were recruited via posted flyers in mammography clinics at the Lynn Sage Comprehensive Breast Center at Northwestern Memorial Hospital. Women were eligible to participate if they: 1) had no personal history of heart disease or stroke; 2) had no personal history of insulin dependent diabetes (Type 1 or Type 2) or taking oral hypoglycemic medications; 3) agreed to wear an activity monitor during the entire time they were awake (except for bathing); 4) had a screening mammogram within one month prior to enrollment at the Lynn Sage; 5) had no personal history of cancer (except non-melanoma skin cancer); 6) were not currently pregnant or lactating; 7) and did not have any physical condition limiting physical activity levels. The study was approved by the Northwestern University and Pennington Biomedical Research Center Institutional Review Boards. All participants signed an informed consent prior to participation.

### Measures

#### Demographic questionnaire

Women completed a questionnaire that included information on leisure time physical activity, race/ethnicity, education, family income, personal and family medical history, ages at menarche and at menopause, menopausal status, and alcohol and tobacco use.

#### Body mass index (BMI)

Weight was measured using a calibrated hospital scale with participants wearing no shoes, and heavy outer clothing removed. Height was measured using a stadiometer with participants wearing no shoes. Weight was recorded to the nearest 0.25 pounds and height to the nearest 0.25 inches. BMI was calculated as the weight (kg) divided by the height squared (m^2^).

#### Activity monitor and determination of sedentary and ambulatory activities

Participants wore the activPAL monitor (PAL Technologies, Glasgow, United Kingdom) for seven days. The activPAL employs both accelerometer and inclinometer functions for objectively assessing sedentary and ambulatory activities. The activPAL has been validated for the measurement of sitting, standing, and walking [25-27].

The time spent sedentary, standing, stepping, and total steps taken were summarized for each day using the raw Excel files generated by the activPAL software (version 5.9.1.1). Step rate was determined by dividing steps by the stepping duration. The guidelines call for accumulating 150 minutes/week of moderate-intensity aerobic exercise-like activity (most commonly brisk walking) in at least 10 min bouts [1]. Prior work has shown that a step rate ≥ 100 steps/min corresponds to approximately ≥3 metabolic equivalents (METS) [28-32], which is the minimum intensity accepted by the guidelines. We allowed for two minutes below 100 steps/min in the 10 min bout, as recommended by a recent survey of physical activity in the United States [33]. Consequently, activity that was sustained for at least eight out of 10 min and was done at a rate of at least 100 steps/min was termed “guideline defined sustained MVPA” when possible in the text. Non-exercise (intermittent) stepping was calculated as the total stepping time minus the time spent in guideline defined MVPA.

Participants were instructed to put the monitor on upon wakening each day and to remove the monitor before going to sleep. Thus, non-wear time was assumed to be during lying down in bed. This time was determined with the participants’ logs (see Procedure) for “time on” and “time off” and the raw Excel files generated by the activPAL software. This non-wear time, assumed to be lying in bed, was subtracted from total sedentary time in order to calculate sitting time.

Participants were asked to wear this monitor for seven days. Only days in which the monitor was worn for at least 10 hours were considered as valid. Likewise, to be included in the analyses, a participant had to achieve at least four valid days of data collection. We calculated weekly exercise for those with less than seven valid days by multiplying their mean daily exercise duration during valid days by seven.

In figures and elsewhere for sake of brevity, the word “exercise” is used to mean “guideline defined sustained MVPA”. Also, this is consistent with what is understood by this public as “exercise” and is currently being used in public health messages, such as the ongoing national “Exercise is Medicine” campaign by the American College of Sports Medicine. However, we acknowledge that sometimes researchers prefer not to use the term “exercise” in studies quantifying sustained MVPA with objective accelerometry, because an activity monitor does not address the subjective intent of the user to promote health [1].

#### Procedure

These women interested in participating in this study underwent a telephone screening to determine study eligibility. Participants who were eligible came to our research clinic to sign the informed consent, completed the demographic questionnaire, had their height and weight measured, and received instruction for how to wear the activity monitor.

The activPAL monitor was worn attached to the front of the right thigh, midway between the knee and hip according to the manufacturer’s instructions. The activPAL was secured to the skin with Hypafix Retention Dressing Tape (Smith & Nephew, London, United Kingdom). Research staff demonstrated the correct position of the monitor and use of adhesive tape while participants were present in the clinic. In addition, participants were sent home with an instruction sheet that reiterated the key points about placement of the monitor, as well as reminders about when to put on and take off the monitors.

Participants were told that the purpose of the study was to assess how much time, in general, women spend each day engaged in physical and sedentary activities. Consequently, they were instructed to maintain their normal daily routine and monitors were not to be worn during vacations or periods of illness. Participants were instructed to begin wearing the activity monitor immediately upon getting out of bed and to wear the monitor for the entire day, with the exception of bathing. They were asked to continue wearing the monitor if watching TV or reading in bed before going to sleep and to remove the monitor only once they were ready to turn out the lights for sleep. In addition, participants were asked to keep a log of the time they put on and took off the monitor each day. Finally, participants were called the morning after their clinic visit to answer any remaining questions about wearing the monitor. As compensation for their time, participants received $50 at the end of their clinic-based study visit and an additional $50 when the activity monitor was returned.

#### Statistical analyses

Means (M), standard deviations (SD), and frequencies were calculated for demographic variables. For between persons analyses, participants were divided into three groups based on total accumulated weekly sustained MVPA: 1) those engaging in None/Low levels of guideline defined sustained MVPA, accumulating < 60 min/week of MVPA [N = 32]; 2) those engaging in Intermediate levels of guideline defined sustained (accumulated in at least 10 min bouts) MVPA, accumulating some MVPA but not meeting recommendations (i.e., these women were accumulating 60–149 min/week of MVPA) [N = 25]; and 3) those Meeting or Exceeding recommendations by accumulating ≥ 150 min/week of guideline defined sustained MVPA [N = 34]. Based on this grouping, the average time in sustained MVPA, should differ between the three groups even after controlling for age. To confirm this, analysis of covariance (ANCOVA), adjusting for age, was conducted to determine whether the three activity groups differed significantly in min/week of sustained MVPA. Next, two separate ANCOVA were conducted, one adjusting for age and another adjusting for both age and wear time, to determine if the three groups differed in sitting time, time spent standing, and in non-exercise step time.

Next, we were interested in examining daily variation in sustained MVPA and sitting time within each individual. We aimed to investigate whether women spend more time sitting on days that they spend less time in sustained MVPA. Based on the American College of Sports Medicine’s recommendation of achieving 30 min of sustained MVPA on all or most days per week [34], we classified each valid day of data as a sufficient MVPA day (defined as accumulating ≥30 min of guideline defined sustained MVPA) or an insufficient MVPA day (defined as accumulating <30 min of guideline defined sustained MVPA) for each person. Next, we calculated average sitting, standing, non-exercise stepping, and MVPA for both types of days for each person. To ensure that any differences noted were not a result of differences in wear time between sufficient and insufficient MVPA days, we conducted a paired *t*-test for wear time as well. Finally, we conducted paired *t*-tests to examine potential differences in sitting time, standing time, and non-exercise stepping time on sufficient MVPA, as compared to insufficient MVPA, days.

Age-adjusted Pearson’s correlations were conducted on the full sample (N=91) to examine relationships among sitting and ambulatory variables of interest (e.g., time spent in guideline defined sustained MVPA, time spent sitting, time spent standing, etc.). A p-value of < 0.05 was considered as statistically significant. Statistical analyses were conducted using SPSS Statistics version 20 (Chicago, IL).

## Results

Complete activity monitor data were available for 91 of the 100 women enrolled in this study. The women wore the monitors for an average of 14.9 hrs/day (SD = 1.1) and 88% of the women had 6–7 valid days of monitor data and 95% had at least one weekend day. Demographic data and mean (SD) for BMI, guideline defined sustained MVPA, sitting, and ambulatory behaviors are presented in Table 1. As can be seen in Table 1, average sustained MVPA, as assessed with the activPAL, was ~150 min/week. This was also consistent with a relatively high mean step count of approximately 10,000 steps/day. However, in spite of this enhanced time spent in MVPA, the majority of waking time each day (~ 63% of day) was spent sitting.

**Table 1:** Demographic variables for study participants (N=91)

**Variables**	**M(SD) or %**
Age (year)	53 (9.0)
Race	
Caucasian	78%
African American	13%
Other	9%
Education	
High School graduate	7%
Some College	17%
College graduate	37%
Graduate School	39%
Menopausal Status	
Pre-Menopausal	49.5%
Post-Menopausal	50.5%
BMI (kg/m^2^)	26.9 (6.3)
Exercise (min/week)	146.0 (144.7)
Sitting (hours/day)	9.1 (1.7)
Standing (hours/day)	3.9 (1.2)
Time Stepping (hours/day)	1.9 (.6)
Step Count (steps/day)	9856.9 (3795.4)

### Between-persons analyses

**Figure 1:**
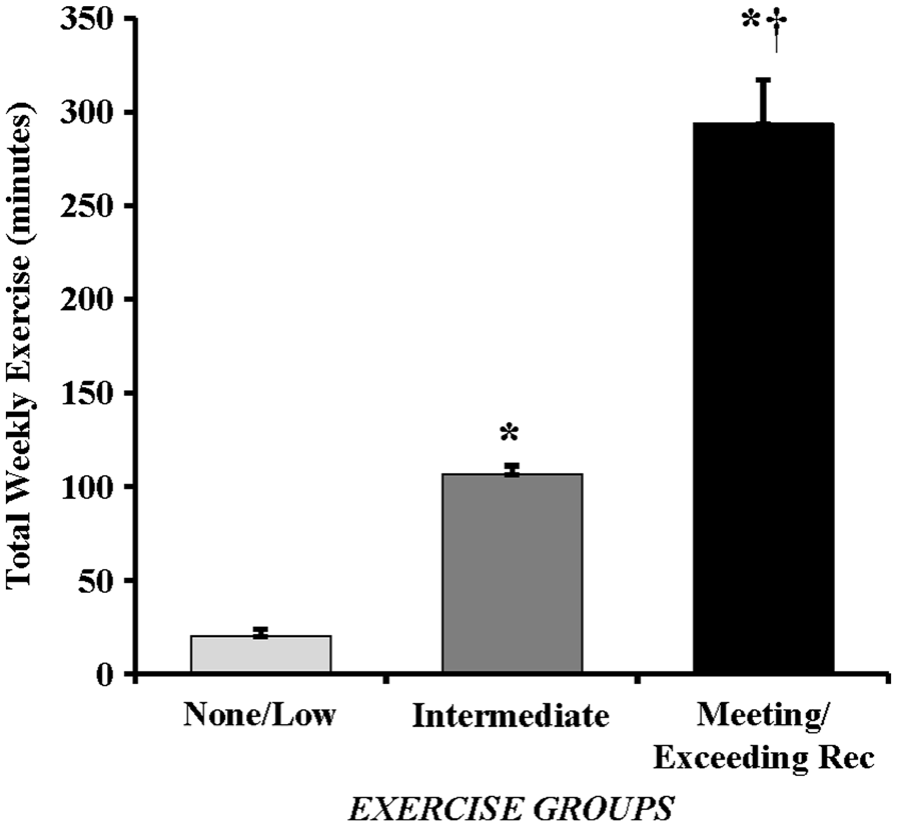
**The accumulative weekly time spent engaged in physical activity as recommended in the federal guidelines for aerobic exercise. ** Women were stratified to represent 3 levels of aerobic exercise duration: 1) *None/Low Exercise *, accumulating < 60 min/week of exercise; 2) *Intermediate *, accumulating some exercise but not meeting recommendations (i.e., accumulating 60–149 min/week of exercise) and, 3) *Meeting or Exceeding Exercise Recommendations * by accumulating ≥ min/week of sustained moderate-vigorous physical activity. Values are expressed as mean with SEM bars. * p<0.001 vs. *None/Low Exercise *, † p<0.001 vs. *Intermediate. *

**Figure 2:**
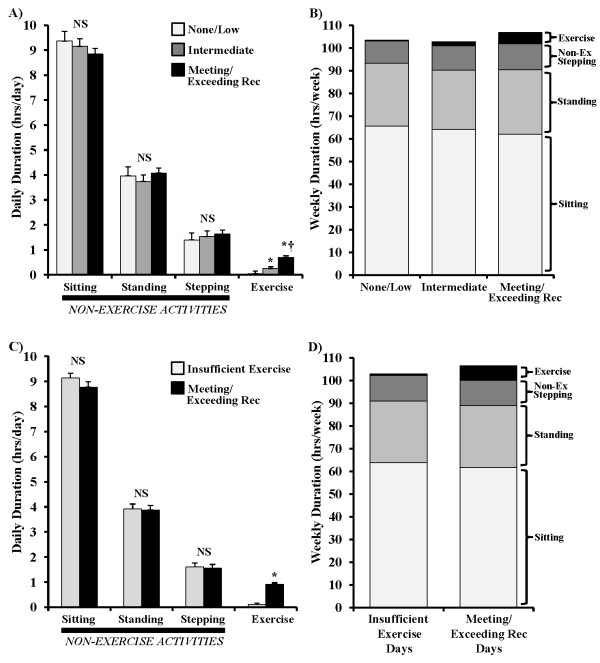
**Time spent sitting, standing, incidental stepping when not exercising (non-exercise stepping), and exercising as defined by the federal physical activity guidelines. ** Panels A and B illustrate the results for a cross-sectional comparison between subjects for the mean daily duration of each behavior (**A**) and the sum of all behaviors accumulated over an entire week (**B**) for the 3 groups stratified by time spent exercising. Panels C and D illustrate the within subject analysis results comparing the days that subjects had Insufficient exercise (<30 minutes) compared to days where they perform at least 30 minutes of aerobic exercise. Values are expressed as means with SEM bars. * p<0.001 vs. *None/Low Exercise or Insufficient Exercise *, † p<0.001 vs. *Intermediate. *

As shown in Figure 1, the three groups were statistically significantly different on min/week of sustained MVPA (p <0.001). Age-adjusted results showed significantly lower exercise in those accumulating None/Low levels of sustained MVPA (Group 1) (M = 20.1 min, SD = 21.4) than the Intermediate MVPA group (Group 2) (M = 106.5 min, SD = 26.1) and the Meeting/Exceeding recommendations for sustained MVPA group (Group 3) (M = 293.7 min, SD = 130.8), (both p≤ 0.001). Similarly, Group 2 had significantly lower MVPA than Group 3 (p<0.001). When examining sitting and ambulatory activities across these categories, there were no significant differences among the three groups for time spent standing, time spent in intermittent stepping, or time spent sitting (all p>0.1) (Figure 2A). Additional adjustment for wear time (in addition to age adjustment) did not alter these findings. Irrespective of time spent in guideline defined sustained MVPA, this MVPA represented only a small portion of total weekly wear time for each group (Figure 2B).

**Figure 3:**
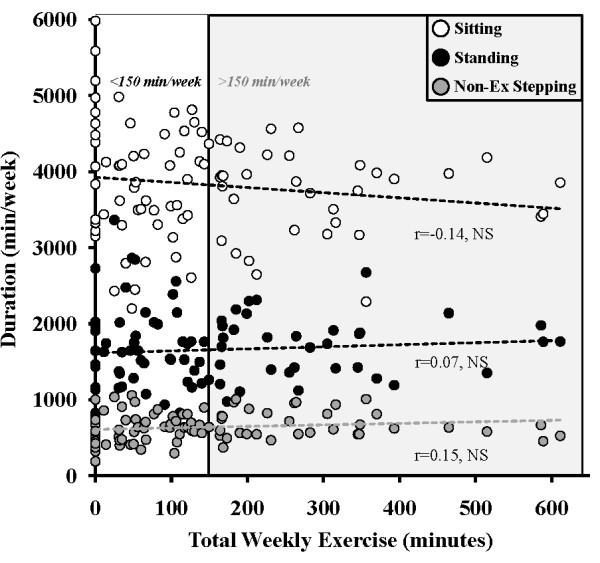
Relationship between the total time spent engaged in exercise as defined in the federal guidelines for sustained physical activity with the 3 non-exercise behaviors, including sitting, standing, and intermittent non-exercise stepping.

Across all participants, time spent in guideline defined sustained MVPA was not significantly correlated (p>0.05) with time spent standing (r = 0.07), in intermittent stepping (r = 0.15), or sitting (r = − 0.14) (Figure 3). This was consistent with the conclusion from the preceding group analyses that time spent in sustained MVPA is not related to sedentary and low-intensity ambulatory behavior (Figure 2A). However, sitting was inversely related to both standing (r= −0.74, p<0.001) and intermittent stepping duration (r= −0.62, p<0.001).

### Within-persons analyses

There were 58 women, of the 91 in the study, who had at least one sufficient MVPA day and at least one insufficient MVPA day and were included in the within-persons analyses. The demographic characteristics of this subset of women were quite similar to the larger sample. These 58 women averaged 53 years of age (SD = 8) and they were predominantly Caucasian (83% White, 10% African American). Half were post-menopausal and the BMI averaged 25.5 kg/m^2^ (SD = 4.4). The women were highly educated, with the majority (95%) having a greater than high school education.

These women averaged 2.8 (SD = 1.6) sufficient MVPA days and 3.9 (SD = 1.6) insufficient MVPA days during the monitoring period. Hours per day that the monitor was worn did not differ significantly (p>0.1) between sufficient (M = 15.0 hrs, SD = 1.3) and insufficient MVPA days (M = 14.7 hrs, SD = 1.1). However, as would be expected, the minutes of guideline defined sustained MVPA did differ significantly p< 0.001 on sufficient (M = 54.7 min, SD = 17.8) versus insufficient MVPA days (M = 5.7 min, SD = 6.6), as did daily mean step rate, an indicator of overall walking intensity (Sufficient Day: M = 96.6 steps/min, SD = 8.5, Insufficient Day: M = 82.8 steps/min, SD = 8.0; p<0.001). Figure 2C demonstrates that, while the difference in average time spent in sustained MVPA between the two types of days was ~ 50 min, there were no significant differences (all p>0.05) between sufficient and insufficient exercise days for standing time (3 min delta, NS), intermittent stepping time (3 min delta, NS), sitting time (18 min delta, NS), or non-wear time (18 min delta, NS).

The best physical activity/inactivity correlate of the difference in exercise time between sufficient and insufficient exercise days was the difference in non-exercise stepping between sufficient and insufficient exercise days (r = −0.20, p = 0.14). This indicates that those with the greatest increase in exercise duration tended to have the largest decrease in non-exercise stepping. The change in sitting time (r = −0.09, NS) and change in non-wear time (r = 0.16, NS) were even weaker correlates with the change in sustained MVPA duration.

## Discussion

This is the first study to examine the relationships between the type of sustained MVPA defined by the Physical Activity Guidelines for Americans and sitting, using objective measures. The results of this study support the hypothesis that exercise and sedentary behaviors (e.g., sitting) are independent classes of behavior [3,4,19,35]. These findings indicate that time spent sitting is not related to time spent in guideline defined sustained MVPA and that, between and within individuals, variations in this MVPA are not significantly associated with sitting, standing, or intermittent stepping time. Our study sample included a relatively large number of women meeting and exceeding the current activity recommendations for MVPA, with the mean of the entire sample averaging 146 min of MVPA/week. In spite of this enhanced time spent in MVPA (as compared to those in the general population), women in our study still spent the majority of their waking day (~63% of their waking hours) sitting. Further, time spent in sustained MVPA comprised only a small fraction of their waking time each day (~2%) and the time spent in MVPA appears to displace only a very small and non-significant amount of time spent in the domains of sleeping and sitting.

We are unaware of any other study that used direct and objective assessment measures from accelerometry and inclinometry to determine the relationship between sedentary behaviors and sustained MVPA (as defined by consensus recommendations of at least 10 minute bouts). However, there are studies that examined the relationship between total accumulated time spent in MVPA (irrespective of bout duration) and total time the body is accelerating below 100 counts per minute [22-24]. While those studies generally show a strong inverse relationship of total low-intensity physical activity with sedentary behavior, they also consistently found low to moderate correlations (r= −0.27 to −0.66) of sedentary behavior with total accumulated MVPA. However, prior work has not specifically focused on the relationship between sustained MVPA performed in at least 10 minute bouts and sedentary time, as we do in the present study. Importantly, in the US population, on average, only about 30% of weekly MVPA is performed in bouts that are of at least 10 min in duration [33]. Thus, previous studies reporting the relationship between sedentary duration and total accumulated MVPA were most likely examining predominantly intermittent MVPA that is not counted toward the weekly goal of 150 min, as defined by the current guidelines. This would include activities like taking out the trash or walking from the parking lot to the store [1,34]. Because those previous studies using accelerometry did not specifically assess activity duration as defined by the guidelines, one cannot draw conclusions about the relationship between guideline defined sustained MVPA and sedentary behavior.

While hip worn accelerometry provides valuable objective information beyond self-report, an advantage of accelerometers with inclinometry, as in the present study, is that they extend the insights to posture (sitting). A recent study using direct observation, in combination with the activPAL and Actigraph monitors, has shown that while the activPAL agrees well with the direct observation of sitting (r=0.94), the hip worn accelerometer (Actigraph), with a 100 counts/min cutpoint, correlates less well with direct observation of sitting (r=0.39). Furthermore, the hip-worn accelerometer was not able to detect a change in sitting behavior caused by a sitting reduction intervention, while the activPAL captured this change [25]. Therefore, the present study is the first, to our knowledge, that used a validated objective measure of sitting to determine if sitting is a distinct behavior or if it is related to the amount of guideline defined sustained MVPA one performs.

There are also many studies that used self-report to examine the association between sedentary behavior and total sustained MVPA. Generally, these studies did not examine sitting over the entire day, but rather focused on either specific sedentary behaviors, like TV watching, or on assessing specific periods of the day, such as leisure time sitting only [8,9]. In a recent study that attempted to assess the relationship between total daily sitting and physical activity in 20 nations using the IPAQ, Bauman et al. reported that there was an inverse relationship between self-reported total MVPA and self-reported sitting [6]. They noted that this finding disagreed with most other studies and speculated that this was due to the IPAQ targeting all domains of physical activity instead of just specific periods of the day like leisure or occupational sitting only. These results are difficult to compare to our current findings given the self-report nature of the measure in that study and the fact that sitting may be under-reported and physical activity over-reported. Proper et al. also used the IPAQ to assess sitting and sustained total MVPA in 1048 Australian adults. They reported an inverse relationship between physical activity (i.e., putatively sustained MVPA) and total daily sitting time but not between MVPA and leisure sitting duration [7]. A point of concern in both studies is that the IPAQ sitting question (i.e., how much one typically sits in the previous seven days) has not been validated against an objective measure of posture like the activPAL or against direct observation. Further, it only weakly agrees with the Actigraph sedentary cutpoint (<100 cts/min) method, which itself is not a strong measure of sitting behavior [25].

Our data show that there was no difference in sitting time between those who met or exceeded the 150 min/week recommendation (far more than the general American population [36]) and the most inactive women (Figure 2A). In addition, there was not even a significant trend, with the women who spent 450–600 min/week in sustained MVPA still sitting ~550 min each day (Figure 3). Thus, the results of the present study clearly show that regardless of the amount of sustained MVPA accumulated, with some women accumulating 4-times the 150 min/week benchmark (Figure 3), more time spent in sustained MVPA is not associated with reduced sitting time.

Many people sit so much (~60-80 hrs/week) that it is impractical to replace significant amounts of total daily sitting time with the type of sustained MVPA recommended for the public. We found that variations in sustained MVPA do not significantly correlate with sitting time, and thus other approaches are necessary for reducing sedentary behavior. This has been alluded to in prior studies that implemented interventions aimed at increasing MVPA but which saw no concurrent decrease in self-reported sitting time [37,38]. However, our data do show that sitting time was significantly and inversely related to intermittent and predominantly low-intensity upright activities. This has relevance to previous research indicating that even LIPA is apparently important for human health [15] because of potent molecular responses related to low-intensity contractile activity locally and specifically caused by postural skeletal muscle [17,18].

There are several strengths and limitations to the current study that should be noted. This is the largest study to date to utilize valid and objective measures (inclinometry) of sitting time and guideline defined sustained MVPA in a cohort of middle and older-aged adult women. In addition, we were well-positioned to examine our study hypothesis as our sample included many women meeting and exceeding the federal physical activity recommendations of 150 min/week of sustained MVPA. Consistent with the Physical Activity Guidelines for Americans, the MVPA bouts of at least 10 minutes were quantified. The determinants, or at least the physiology, of this type of sustained activity is different than the more intermittent and short bouts of activity. With respect to limitations, this was a cross-sectional study of women only and we cannot determine causal relationships between sitting time and time spent in sustained MVPA, nor can we generalize to men or other populations. It is also plausible that there are other unmeasured factors that could influence the relationships we observed in this study.

## Conclusions

Our findings suggest that sitting is not the behavioral equivalent of exercising too little. The ultimate goal for this emerging field of inactivity physiology, whether at the level of the individual or with respect to public health efforts, is to reduce total sedentary time and to increase the number of breaks in sedentary time [39]. Thus, a primary concern for this field should be identifying how to make a very sedentary public much less sedentary. Almost every sector of our society sits for prolonged periods almost every day. The healthy and relatively very active women in the present study (average step count was ~10,000 steps/day and high MVPA) sat 9 hrs/day, which is more than the average adult sleeps [40]. Consequently, sitting is now more abundant than sleeping, which is likely an important milestone in human history. Our data suggest that time spent in recommended MVPA does not replace significant periods of sitting time. Thus, public health recommendations and interventions aimed at increasing MVPA (i.e. "exercise") are unlikely to impact how much time people spend sitting. Consequently, our data support the emerging contention that there is a need for new and separate recommendations aimed at reducing sitting time. Additional studies aimed at confirming our present results in other populations (ie., males, children, young, and old) will strengthen the conclusions of this study. This would also lead to a more thoroughly investigated and supported revision to the guidelines for physical activity and health.

## Competing interests

There are no competing interests to disclose for any of the manuscript authors.

## Authors’ contributions

LC, SG, TZ, & MH participated in the design of the study, collection of data, statistical analyses, drafting and critical revision of manuscript. EV & DT participated in collection of the data, drafting and critical revision of the manuscript. JS participated in the statistical analyses and interpretation of the data, drafting and critical revision of the manuscript. All authors read and approved the final manuscript.
